# Data for benchmarking low-cost, 3D printed prosthetic hands

**DOI:** 10.1016/j.dib.2019.104163

**Published:** 2019-06-22

**Authors:** Farah Alkhatib, John-John Cabibihan, Elsadig Mahdi

**Affiliations:** Mechanical and Industrial Engineering Department, Qatar University, Doha, 2713, Qatar

**Keywords:** Grasping force measurements, Finite element modeling, Material properties

## Abstract

In this article, three different data sets are presented to evaluate a representative of openly accessible 3D printed prosthetic hand. The first data set includes grasping force measurements of human hand and low-cost 3D printed hand. Three grasping functions were evaluated, spherical, cylindrical, and precision grasps. The experimental test was performed using a wearable tactile sensor. The second data set includes the numerical analysis of prosthetic fingers made from Acrylonitrile Butadiene Styrene (ABS) and Polylactic Acid (PLA) materials under different carrying loads. The numerical analyses were carried out by LS-DYNA software. The files can be used for the prosthetic fingers’ evaluation and for the selection of suitable material. The third data set includes the experimental tensile test of ABS and PLA materials. The mechanical properties were calculated from the results, which can be used in the design and fabrication of products from these materials. All the datasets are available from Harvard Dataverse: https://doi.org/10.7910/DVN/GCPAIL.

**Table:** Specifications table

Subject area	Medical, engineering
More specific subject area	Failure analysis of available prosthetic hand.
Type of data	Datasets, FEM, tables.
How data was acquired	• Tactile sensor was fitted on human and prosthetic hands for grasping forces measurements. • Numerical software. • Extensometer fixed on the tensile specimen.
Data format	Tabular data, finite element input files.
Experimental factors	• Grasping forces measurement. • Numerical modeling. • Mechanical properties of plastic materials.
Experimental features	• The collected grasping force data were used for comparison between human and prosthetic hand for same objects. • Numerical modeling of 3D printed prosthetic finger from open-source design. • ABS and PLA specimens were subjected to experimental tensile test according to ASTM D638.
Data source location	Qatar University, Doha 2713, Qatar.
Data accessibility	Data are available online at the Harvard Data in Brief Dataverse https://doi.org/10.7910/DVN/GCPAIL

**Table:** 

**Value of the data** • The first collected data set can be used for evaluation the low-cost 3D printed hand grasping functions compared to the human hand. • The second data set can be used for evaluation of the material 3D printed prosthetic hands and the calculation of the lifetime of the prosthetic hands. • The third data set can be used in designing and manufacturing products made from Acrylonitrile Butadiene Styrene (ABS) and Polylactic Acid (PLA) materials.

## 1 Data

The widespread availability of desktop 3D printers has enabled the fabrication of low-cost 3D printed hands [1], [2], [3]. With this development, it became necessary to investigate the functional capabilities and the limitations of the typical materials used for this type of artificial hands [4], [5]. In this article, three different data sets are presented. The first set consist of raw data files for grasping forces measurements. The data were collected from an experimental test performed on human hand and low-cost 3D printed hand. The second set consist of finite element model for the failure analysis of low-cost 3D printed hand index finger. The folder contains k files of ABS and PLA finger under different carrying loads. The third data set consist of experimental tensile test of ABS and PLA materials. The folder contains the load-displacement data for both materials with respect to time.

### 1.1 Detailed description of the grasping forces data files

The data files of the experimental grasping forces measurements contain the human hand and prosthetic hand grasping forces data. Three objects were used to simulate each grasping function, a plastic ball was used for spherical grasping, a 330-ml water bottle was used for cylindrical grasping, and a wooden cube was used for precision grasping (grasping forces.xlsx).

### 1.2 Detailed description of the finite element model (FEM) of a 3D printed prosthetic hand

Finite Element Analysis has been an effective tool for analyzing contact interactions between artificial fingers and various objects [6], [7], [8], [9], [10], [11], [12]. For this data set, the index finger of the low-cost 3D printed prosthetic hand was subjected to numerical failure analysis using LS-DYNA software (mmps R8.1.1, Livermore Software Technology Corporation (LSTC), USA) [13]. The k files with full description are presented. The ABS and PLA versions of the fingers with different carrying load capacities are available (FEM raptor reloaded index finger.zip).

### 1.3 Detailed description of tensile test for ABS and PLA materials

The mechanical properties of the printable ABS and PLA materials were obtained from the experimental tensile test (Tensile_test.zip). ABS and PLA mech properties.xlsx contains the columns that represent the load and displacement data for both ABS and PLA materials.

## 2 Experimental design, materials, and methods

### 2.1 Grasping force experiment

A wearable tactile sensory system (FingerTPS, Pressure Profile Systems, CA, USA) was used for the grasping force measurements of different grasping functions for the human hand and a low-cost 3D printed prosthetic hand. This sensor system was reliably used in earlier tactile experiments in prosthetics and biomechanics [5], [14], [15]. Spherical, cylindrical, and precision grasping functions were tested for each hand for 30 times over the same period using the test protocol similar to [16]. The volunteer was asked to wear the sensors and apply the grasping forces on different objects. For the spherical grasp, a 10-g plastic ball was used to measure the grasping forces. The cylindrical grasp was analyzed using a 330-ml water bottle. For evaluating the precision grasp, a 50-g wooden cube was used. All the mentioned grasping functions were reapplied on the prosthetic 3D printed hand by controlling the wrist joint manually. Finally, before taking any measurements, each finger was calibrated individually using the provided reference calibration sensor.

**Fig. 1:**
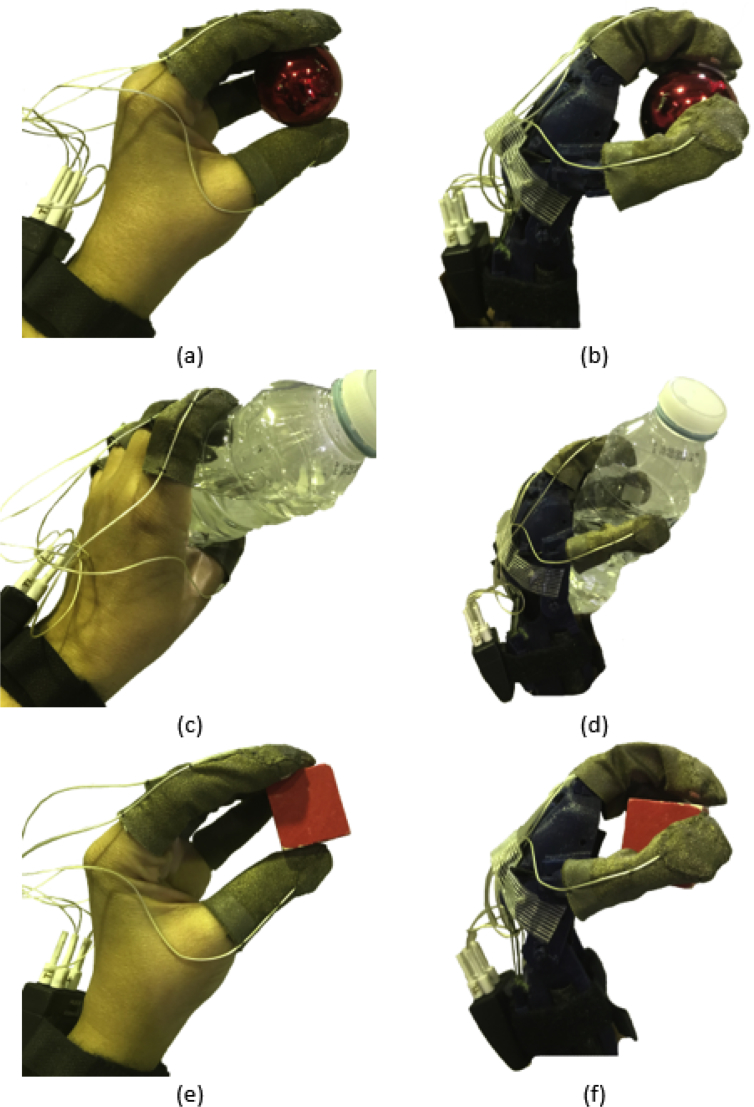
Experimental setup for different grasping functions performed by human hand and 3D printed prosthetic hand. (a) spherical grasp of human hand, (b) spherical grasp of prosthetic hand, (c) cylindrical grasp of human hand, (d) cylindrical grasp of prosthetic hand, (e) precision grasp of human hand, and (f) precision grasp of prosthetic hand.

In the spherical grasping of human hand (Fig. 1a), the thumb, index, and middle fingers are involved in this function. Only the thumb and index fingers were involved in the same grasp of the prosthetic hand (Fig. 1b). For the human cylindrical grasp, the five fingers were in contact with the bottle (Fig. 1c). The thumb, index, middle, and ring fingers have the contact with the bottle in the prosthetic cylindrical grasp (Fig. 1d). In the precision grasp of human hand, the thumb, index, middle, and ring fingers were used for the grasping (Fig. 1e). For the prosthetic hand, the thumb, index, and middle fingers have contributed for grasping the wooden cube (Fig. 1f).

### 2.2 Finite element modelling description

**Fig. 2:**
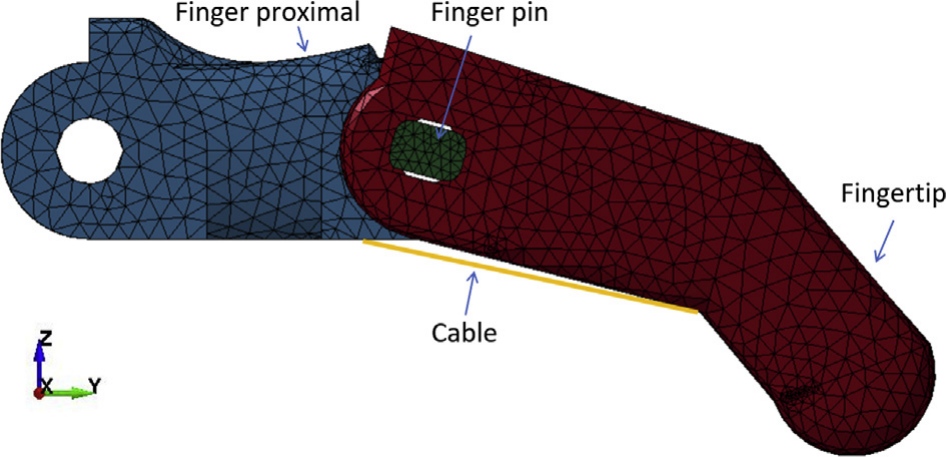
Finite element modelling of the index finger.

Fig. 2 shows the index finger presented in LS-DYNA software. Full description of the used keywords are described below.

#### 2.2.1 Elements

• 3D quadratic tetrahedron solid elements were used to model the fingertip, finger proximal and finger pin. Each element has four nodes and one nodal rotation to eliminate the probability of rotational deformation.
• Beam elements were used to model the cable because the cable has constant cross-sectional properties. The beam elements consist of three nodes in three-dimensional space. Two nodes are for identifying geometry and the third node is for the orientation of the beam element.
• Discrete element: was used to model the elastic cable, with one degree of freedom and two nodes. This discrete element has a spring behavior to simulate the elasticity of the elastic cable.

#### 2.2.2 Materials

• MAT_PIECEWISE_LINEAR_PLASTICITY (MAT_024) was used to model the ABS and PLA fingertip, finger proximal, and finger pin. This material allows to define an elasto-plastic behavior. These materials are known for their compound elastic and plastic behavior after reaching the yield stress of the material, which is unlike metals that undergo plastic deformation after their yield stresses.
• MAT_PLASTIC_KINEMATIC (MAT_003) was used to model the tension cable with very low strain rate, since this non-elastic cable has almost no deformation with respect to time.

#### 2.2.3 Loading

• LOAD_POINT keyword was used to apply the tension force on one node of the cable. The fingers were subjected to 5 N, 15 N, and 25 N.

### 2.3 Experimental tensile test

**Fig. 3:**
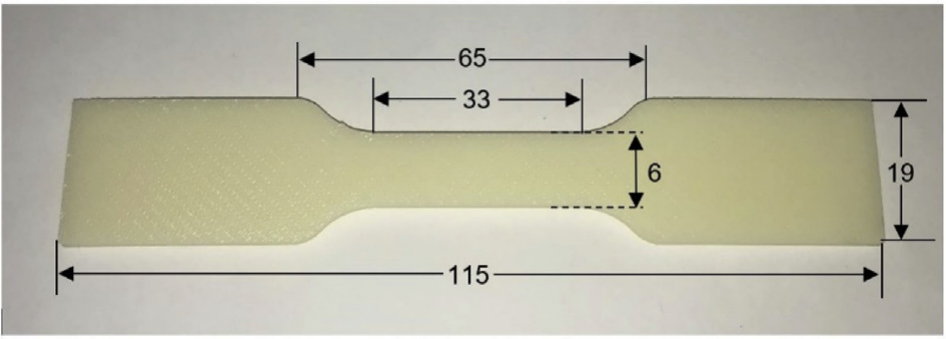
A 2 mm thick ABS 3D printed sample with dimensions in mm (ASTM D638).

Experimental tensile test by 50 kN Instron machine (5969 Series Universal Testing Systems, Instron, USA) was performed at 5 mm/min for ABS and PLA materials. The samples of ABS and PLA were printed according to ASTM D638 standard at 215 °C with 0°/90° filament orientation (Fig. 3). Table 1 presents the results of the experimental tensile test.

**Table 1:** The mechanical properties obtained from the tested 3D printed ABS and PLA samples.

	Mass density [g/cm^3^]	Young's modulus [GPa]	Ult. tensile stress [MPa]	Failure strain [%]
ABS	1.10	1.40	32.00	1.05
PLA	1.30	3.90	54.00	2.20
